# Measuring abortion-related mortality: challenges and opportunities

**DOI:** 10.1186/s12978-015-0064-1

**Published:** 2015-09-16

**Authors:** Caitlin Gerdts, Ozge Tunçalp, Heidi Johnston, Bela Ganatra

**Affiliations:** Ibis Reproductive Health, Oakland, California USA; UNDP/UNFPA/UNICEF/WHO/World Bank Special Programme of Research, Development and Research Training in Human Reproduction (HRP), Department of Reproductive Health and Research, World Health Organization, Geneva, Switzerland

## Abstract

Two recent efforts to quantify the causes of maternal deaths on a global scale generated divergent estimates of abortion-related mortality. Such discrepancies in estimates of abortion-related mortality present an important opportunity to explore unique challenges and opportunities associated with the generation and interpretation of abortion-related mortality estimates. While innovations in primary data collection and estimation methodologies are much needed, at the very least, studies that seek to measure maternal deaths due to abortion should endeavor to improve transparency, acknowledge limitations of data, and contextualize results. As we move towards sustainable development goals beyond 2015, the need for valid and reliable estimates of abortion-related mortality has never been more pressing. The post-MDG development agenda that aims to improve global health, reduce health inequities, and increase accountability, requires new and novel approaches be tested to improve measurement and estimation of abortion-related mortality, as well as incidence, safety and morbidity.

Over the last decade, global levels of maternal mortality have decreased [1]. Improved levels of overall health, increased availability of medical abortion, and efforts to improve access to safe abortion and post-abortion-care in some countries may have contributed to reductions in abortion-related mortality. Evaluating the extent of progress however, remains problematic due to challenges in obtaining valid and reliable data and difficulties interpreting existing data.

Two recent efforts to quantify the causes of maternal deaths on a global scale generated divergent estimates of abortion-related mortality. Despite overlapping confidence intervals, the point estimate for the proportion of maternal mortality attributable to abortion from the 2013 IHME estimate (14.9 %, 95 % Uncertainty Interval (UI): 13.5, 17.6) is nearly twice that estimated for the 2003-2012 period by the WHO (7 · 9 %, 95 % UI: 4 · 7,13 · 2) [2, 3]. Indeed, the two papers examined different time periods, employed distinct statistical techniques, and analyzed discrete data sources. Nevertheless, such discrepancies in estimates of abortion-related mortality present an important opportunity to explore unique challenges and opportunities associated with the generation and interpretation of abortion-related mortality estimates.

Estimates of abortion-related mortality are primarily developed from four types of data sources: confidential enquiries, vital registration data, verbal autopsy-- a systematic tool used to collect health information from lay-person informants and assess causes of death [4] and facility-based data sources. National-level confidential enquiries into causes of maternal mortality only occur in a handful of countries. While vital registration systems are considered the gold-standard for mortality measurement, they have been shown to miss up to 30-50 % of all maternal deaths. In addition, 75 % of global births take place in countries without existing vital registration systems [5–7]. For both facility-surveys and verbal autopsies, willingness to participate in studies, misclassification, and underreporting present obstacles to the collection of robust data. In countries where unsafe abortion is common, it is often also legally restricted, and/or highly stigmatized. In these settings, fear of legal or social repercussions, lead women who experience abortion-related complications to be less likely than women experiencing other kinds of pregnancy-related complications to seek care in medical facilities [8–11]. This type of bias--selection bias--results in an absolute undercount of abortion-related deaths (and a relative undercount of abortion-related deaths compared with other causes of maternal deaths) from facility-based data [12].

While verbal autopsy studies may provide some advantages over facility-based data in the estimation of community-level distribution of abortion-related mortality, concerns over selection bias persist: fear of social and legal ramifications may lead family members of women who suffered an abortion-related death to be less likely to participate in studies as compared to family members of women who died from other maternal causes [13, 14]. Additionally, for both data sources, because complications from induced abortion often manifest similarly to other obstetric complications (spontaneous abortion, hemorrhage, sepsis) abortion-related deaths are prone to being misclassified as non-abortion-related maternal deaths, or even as non-maternal deaths—again resulting in absolute and relative underestimates of abortion-related deaths [11]. Finally, in both facility-based and verbal autopsy studies, abortion-related deaths are more likely than the other maternal causes to be classified as “unknown” [15]. By virtue of being classified as “unknown”, abortion-related deaths are misclassified as non-abortion-related deaths, leading to an underestimate of abortion-related deaths as a proportion of all maternal deaths.

In addition to the above-mentioned concerns, there are limitations to what can be interpreted from estimates of abortion-related deaths. First, global estimates of cause-specific maternal death present abortion-related mortality as a proportion of total mortality. Any increase or decrease in abortion-related mortality is relative to other causes and does not necessarily imply that abortions have become safer or less safe with respect to mortality. Second, drawing inference about the safety of abortion from estimates of abortion-related mortality requires a more comprehensive understanding of the circumstances within which abortions take place. Lower or higher levels of abortion-related mortality could be driven by multiple, non-mutually-exclusive factors a) fewer induced abortions taking place, b) induced abortions occurring under safer conditions, c) abortions continuing in high risk circumstances but improvements in post-abortion care result in fewer abortion-related deaths. Taken independently or together, these factors highlight the fact that abortion-related mortality does not happen in isolation, and that both the incidence and safety of abortion must be considered when developing and interpreting estimates of abortion-related mortality [16].

Among the causes of maternal death, abortion is likely the least well measured [17–19], and methodological advances in measuring abortion-related mortality have been slow to develop. While innovations in primary data collection and estimation methodologies are much needed, at the very least, studies that seek to measure maternal deaths due to abortion should endeavor to follow three simple recommendations: 1) Prioritize transparency, 2) Acknowledge limitations of data, and 3) Contextualize results.

**Table:** 

Recommendations for collection and reporting of abortion-related mortality data
**• Prioritize transparency**
*Specify data sources, clearly describe statistical models, and, where possible make data, coding, and publications publically available/open access.*
**• Acknowledge limitations of data**
*Identify potential biases, describe hypothesized direction and magnitude of biases, and convey probable impact on results.*
**• Contextualize results**
*Facilitate interpretation of abortion-related mortality estimates by presenting and interpreting results within a context of abortion safety and incidence.*

The examination of current and historical estimates of abortion-related mortality highlights both the importance of strengthening local and national level systems for the collection of routine health information as well as the importance of programmatic action to eliminate the criminalization of abortion, reduce abortion-related stigma, and increase access to safe abortion. As we move towards sustainable development goals beyond 2015 with a proposed aim of ending preventable maternal mortality (EPMM) [20], the need for valid and reliable estimates of abortion-related mortality has never been more pressing. The post-MDG development agenda that aims to improve global health, reduce health inequities, and increase accountability, requires new and novel approaches be tested to improve measurement and estimation of abortion-related mortality, as well as incidence, safety and morbidity.
